# Differentiation of thyroid nodules on US using features learned and extracted from various convolutional neural networks

**DOI:** 10.1038/s41598-019-56395-x

**Published:** 2019-12-27

**Authors:** Eunjung Lee, Heonkyu Ha, Hye Jung Kim, Hee Jung Moon, Jung Hee Byon, Sun Huh, Jinwoo Son, Jiyoung Yoon, Kyunghwa Han, Jin Young Kwak

**Affiliations:** 1 Department of Computational Science and Engineering, Yonsei University, Seoul, South Korea; 2 Department of Radiology, School of Medicine, Kyungpook National University, Kyungpook National University Chilgok Hospital, Seoul, South Korea; 3 Department of Radiology, Severance Hospital, Research Institute of Radiological Science, Yonsei University College of Medicine, Seoul, South Korea

**Keywords:** Cancer imaging, Head and neck cancer

## Abstract

Thyroid nodules are a common clinical problem. Ultrasonography (US) is the main tool used to sensitively diagnose thyroid cancer. Although US is non-invasive and can accurately differentiate benign and malignant thyroid nodules, it is subjective and its results inevitably lack reproducibility. Therefore, to provide objective and reliable information for US assessment, we developed a CADx system that utilizes convolutional neural networks and the machine learning technique. The diagnostic performances of 6 radiologists and 3 representative results obtained from the proposed CADx system were compared and analyzed.

## Introduction

Advances in high-resolution ultrasonography (US) along with increased access to health check-up services and increased medical surveillance have led to a massive escalation in the number of detected thyroid nodules, especially small thyroid nodules, and thyroid nodules have been detected in up to 68% of adults^1^. US is recognized as the best diagnostic tool for thyroid nodules due to its sensitivity and accuracy. However, US is an operator-dependent and subjective imaging modality^2^. While interobserver variability (IOV) is very low among experienced physicians^3^, poor agreement was documented when US findings of thyroid nodules were interpreted by less experienced physicians^4^.

In order to support the decision-making process of physicians by adding objective opinions, computer-aided diagnosis (CADx) has been introduced and developed over the years^5–7^. CADx provides physicians with second opinions from computational and statistical perspectives so that physicians can refer to the information obtained through CADx and use it as supplementary data to reach their final decision. In conventional CADx systems, feature extraction and classification are common processes. Feature extraction involves extracting information and generating features from original data. Classical techniques for feature extraction are based on mathematical and statistical approaches, and handcrafted features including textural and morphological properties are extracted. Textural features include information such as contrast, coarseness, roughness, and intensity and morphological features include information such as perimeter, circularity, elongation, and compactness^8–10^. Classification integrates the extracted features and then estimates the class of data. Many classifiers are variations of Support Vector Machine (SVM), decision tree, K-nearest neighbor, etc^11^. Both feature extraction techniques and classification methods have been widely used for thyroid US images^5,12–21^.

However, extracting meaningful features often results in loss of good characteristics due to a heavy dependence on problems. Therefore, series of trial and error are required to get optimal results and this in turn can increase operational costs. Deep learning has attracted attention to recent image classification problems by showing outstanding results in the ImageNet Large Scale Visual Recognition Competition (ILSVRC). Early in the 2010s, feature extraction based on deep learning was introduced as big data began to be utilized in the medical field^22–24^.

The deep learning method not only generates non-handcrafted features from original data but also acts as a classifier. Recently, many studies have applied deep learning to medical image analysis. Convolutional Neural Networks (CNNs), a popular deep learning structure, are widely used for this analysis^25^. Typically, good learning processes require big data which are not often available, especially in the medical imaging field. For this reason, we use CNN models trained by huge amounts of data with various classes in a process called transfer learning^26,27^.

Previous studies have applied deep learning methods to the classification of thyroid nodules on US^6,28,29^. Other studies have also focused on CNN-based features and have applied them to existing classifiers^30,31^. In this study, we employed various trained CNNs to combine features and to use them to diagnose thyroid nodules on US through classifiers, and compared the diagnostic performance of CNNs with that of radiologists with various levels of experience.

## Results

We performed 2 machine learning algorithms which were trained with the combined features from 6 pre-trained CNNs to classify thyroid nodules on US images. Representative outcomes were then selected and compared with the diagnostic performances of the 6 radiologists. We first examined the performances of the fine-tuned CNNs. Afterwards, the proposed combinations for CNN-based feature extraction and classifier results were presented and analyzed. Here, accuracy (Acc), specificity (Spe), and sensitivity (Sen) were the three performance evaluation criteria and calculated as follows.

$${\rm{Acc}}=\frac{{\rm{TP}}+{\rm{TN}}}{{\rm{TP}}+{\rm{TN}}+{\rm{FN}}+{\rm{FP}}},\,{\rm{Spe}}=\frac{{\rm{TN}}}{{\rm{TN}}+{\rm{FP}}},\,{\rm{Sen}}=\frac{{\rm{TP}}}{{\rm{TP}}+{\rm{FN}}},$$

where TP (true positive) is the number of nodules correctly predicted as malignant, TN (true negative) the number of nodules correctly predicted as benign, FP (false positive) the number of nodules inaccurately predicted as malignant, and FN (false negative) the number of nodules inaccurately predicted as benign. Acc, Spe, Sen and AUC were expressed as values multiplied by 100 in the tables. The diagnostic results with 150 test images interpreted by six radiologists who had different levels of experience are presented for comparison (see Table 1).

**Table 1:** Diagnostic performances of radiologists and CNNs.

	TP	FN	FP	TN	Accuracy	Specificity	Sensitivity
Faculty 1	91	9	24	26	78 (70.64, 83.93)	52 (38.63, 65.08)	91 (83.58, 95.26)
Faculty 2	76	24	2	48	82.67 (75.8, 87.89)	96 (85.32, 99)	76 (66.75, 83.32)
Fellow 1	63	37	3	47	73.33 (65.81, 79.71)	94 (82.92, 98.06)	63 (53.19, 71.84)
Fellow 2	65	35	4	46	74 (66.59, 80.25)	92 (80.81, 96.91)	65 (55.23, 73.65)
Resident 1	49	51	6	44	62 (53.97, 69.42)	88 (75.99, 94.44)	49 (39.37, 58.71)
Resident 2	63	37	14	36	66 (58.37, 72.88)	72 (57.53, 83)	63 (53.36, 71.71)
CNN 1	96	4	5	45	94 (88.83, 96.86)	90 (78.03, 95.8)	96 (89.82, 98.49)
CNN 2	94	6	3	47	94 (88.83, 96.86)	94 (82.92, 98.06)	94 (87.27, 97.28)
CNN 3	98	2	7	43	94 (88.88, 96.85)	86 (73.28, 93.23)	98 (92.45, 99.49)

Note. - Data in parentheses are 95% confidence intervals.

TP = true positive; FN = false negative; FP = false positive; TN = true negative; CNN = deep convolutional neural network; AUC = area under the curve.

### Conventional approaches

The conventional CNN results obtained without separating feature extraction and classification processes are presented in Table 2. Furthermore, in Table 3, we presented the performances observed when the features extracted from a single CNN and one of the SVM/RF classifiers were used (details of CNNs and classifiers can be found in Supplementary Information). These results were compared with the results obtained with the proposed method.

**Table 2:** Performances of fine-tuned CNNs.

Net	AlexNet	OverFeat	VGG	VGG-verydeep	ResNet	Inception
Acc	86.7	85.3	86	85.3	84	86.7
Spe	88	86	84	74	86	78
Sen	86	85	87	91	83	91
AUC	90.3	88.4	89.3	90.6	90.5	88.3

**Table 3:** Extended features from a single CNN with/without fine-tuning and classification using SVM/RF: ‘Name’ follows the form ‘extracted layer-classifier’ and # denotes the number of features.

Net	Name	#	Without fine-tuning				With fine-tuning			
			Acc	Spe	Sen	AUC	Acc	Spe	Sen	AUC
AlexNet	fc1-SVM	4096	80.0	80.0	80.0	89.2	87.3	86.0	88.0	91.2
	fc1-RF	4096	85.3	82.0	87.0	88.4	86.0	82.0	88.0	88.7
	fc2-SVM	4096	81.3	80.0	82.0	88.3	84.7	82.0	86.0	90.0
	fc2-RF	4096	84.0	78.0	87.0	86.9	87.3	82.0	90.0	88.3
	fc1fc2-SVM	8192	82.0	80.0	83.0	89.0	85.3	84.0	86.0	90.7
	fc1fc2-RF	8192	86.0	82.0	88.0	86.6	87.3	84.0	89.0	88.8
OverFeat	fc1-SVM	4096	78.7	74.0	81.0	86.7	84.7	82.0	86.0	90.6
	fc1-RF	4096	81.3	78.0	83.0	84.3	87.3	84.0	89.0	89.9
	fc2-SVM	4096	81.3	80.0	82.0	86.8	85.3	84.0	86.0	89.6
	fc2-RF	4096	81.3	74.0	85.0	84.8	88.0	84.0	90.0	88.4
	fc1fc2-SVM	8192	81.3	76.0	84.0	86.6	85.3	84.0	86.0	90.2
	fc1fc2-RF	8192	82.0	72.0	87.0	85.1	88.0	86.0	89.0	89.5
VGG	fc1-SVM	4096	79.3	82.0	78.0	86.5	84.7	80.0	87.0	90.7
	fc1-RF	4096	84.7	80.0	87.0	86.4	89.3	86.0	91.0	90.7
	fc2-SVM	4096	80.7	84.0	79.0	86.1	86.0	82.0	88.0	90.6
	fc2-RF	4096	85.3	80.0	88.0	86.8	88.0	84.0	90.0	90.8
	fc1fc2-SVM	8192	79.3	82.0	78.0	86.2	86.7	82.0	89.0	91.0
	fc1fc2-RF	8192	82.7	80.0	84.0	83.4	88.7	84.0	91.0	90.8
VGG-verydeep	fc1-SVM	4096	84.7	88.0	83.0	91.4	78.0	76.0	79.0	85.8
	fc1-RF	4096	84.0	88.0	82.0	91.1	74.0	76.0	73.0	80.4
	fc2-SVM	4096	84.0	88.0	82.0	91.0	72.0	76.0	70.0	81.2
	fc2-RF	4096	85.3	90.0	83.0	89.9	69.3	74.0	67.0	75.6
	fc1fc2-SVM	8192	84.7	88.0	83.0	91.1	76.0	74.0	77.0	85.9
	fc1fc2-RF	8192	85.3	92.0	82.0	90.6	71.3	74.0	70.0	77.9
ResNet	avg-SVM	2048	84.0	82.0	85.0	89.8	74.7	82.0	71.0	84.7
	avg-RF	2048	85.3	86.0	85.0	90.9	76.7	80.0	75.0	85.6
Inception	avg-SVM	2048	85.3	82.0	87.0	88.3	75.3	70.0	78.0	82.9
	avg-RF	2048	84.7	72.0	91.0	87.4	76.0	68.0	80.0	78.2

As depicted in Table 3, AlexNet, OverFeat, and VGG showed that features extracted from fine-tuned CNNs and SVM or RF classification using these features produced similar or better results than the ones in Table 2. Conversely, VGG-verydeep, ResNet, Inception showed that a SVM/RF classifier associated with features extracted from pre-trained CNNs led to similar or worse results than fine-tuned CNN in Table 2. Taken together, feature extraction techniques based on CNNs combined with SVM/RF classifiers may have worse results than fine-tuned CNNs (Table 2) with deeper layers. Otherwise, there is a possibility that the training dataset was not large enough to tune a huge amount of parameters. Thus, fine-tuning with a small dataset may harm good parameters which can generate useful and objective features. When classifiers were compared, RF often performed better than SVM.

### Feature concatenation

Based on the idea that different structures in CNN will provide different features, we selected effective features for each CNN based on the results shown in Table 3 and concatenated them. We chose CNN features extracted from AlexNet^32^-fc2 with fine-tuning ([A]), OverFeat^33^-fc2 with fine-tuning ([O]), VGG^34^-fc1 with fine-tuning ([V]), VGG-verydeep^35^-fc2 without fine-tuning ([Vv]), ResNet^36^-avg without fine-tuning ([R]), and Inception^37^-avg without fine-tuning ([I]). Table 4 summarizes the results of the selected features. Even though AlexNet, OverFeat, VGG, VGG-verydeep allow self-feature-concatenations since features can be extracted from two different layers in a single net, we decided not to use them due to there being almost no effect with self-concatenation. We expected feature concatenation to improve results compared to when it was not performed (Table 3), so we added a new performance criterion $\tilde{J}$ which is calculated as follows

$$\tilde{J}=\frac{{\rm{Acc}}/{\rm{Spe}}/{\rm{Sen}}\,{\rm{with}}\,{\rm{feature}}\,{\rm{concatenation}}\,{\rm{and}}\,{\rm{classifier}}\,{\rm{SVM}}/{\rm{RF}}}{{\rm{maximum}}\,{\rm{of}}\,{\rm{Acc}}/{\rm{Spe}}/{\rm{Sen}}\,{\rm{with}}\,{\rm{single}}\,{\rm{CNN}}\,{\rm{feature}}\,{\rm{and}}\,{\rm{classifier}}\,{\rm{SVM}}/{\rm{RF}}\,{\rm{in}}\,{\rm{Table}}\,2}.$$

**Table 4:** Selected CNN features: AlexNet-fc2 with fine-tuning [A], OverFeat-fc2 with fine-tuning [O], VGG-fc1 with fine-tuning [V], VGG-verydeep-fc2 without fine-tuning [Vv], ResNet-avg without fine-tuning [R], Inception-avg without fine-tuning [I].

Name	Classifier							
	SVM				RF			
	Acc	Spe	Sen	AUC	Acc	Spe	Sen	AUC
[A]	84.7	82.0	86.0	90.0	87.3	82.0	90.0	88.3
[O]	85.3	84.0	86.0	89.6	88.0	84.0	90.0	88.4
[V]	84.7	80.0	87.0	90.7	89.3	86.0	91.0	90.7
[Vv]	84.0	88.0	82.0	91.0	85.3	90.0	83.0	89.9
[R]	84.0	82.0	85.0	89.8	85.3	86.0	85.0	90.9
[I]	85.3	82.0	87.0	88.3	84.7	72.0	91.0	87.4

The quantity $\tilde{J}$ indicates whether or not the criteria of feature concatenation led to better results than individual criteria. An asterisk (*) indicated performance values of feature concatenations that had a $\tilde{J}$ smaller than 1. Table 5 shows the feature concatenations of two or three CNN features and Table 6 shows the feature concatenations of four or more CNN features, respectively. One can tell from Table 5 that most of the feature concatenations provide improved results compared with individual results, with the word ‘individual’ henceforth indicating results obtained using features from a single CNN in Table 3. With SVM, all results except for [VvI] showed improved accuracy than individual results. Notable results were found when we applied feature concatenations of four or more CNN features. In Table 6, all accuracies and sensitivities improved compared to individual cases. For instance, minimum accuracy and sensitivity was 90.0% and 91.0%, and maximum accuracy and sensitivity was 94.0% and 99.0%, respectively. This shows that accuracy and sensitivity are guaranteed to improve when feature concatenations of more various CNN features are applied.

**Table 5:** Feature concatenation (2 or 3 CNNs) results: $[{{\rm{N}}}_{1},\cdots ,{{\rm{N}}}_{{\rm{k}}}],\,{\rm{k}}=2,3$ denotes feature concatenation using the features from CNNs, ${{\rm{N}}}_{1}$ to ${{\rm{N}}}_{{\rm{k}}}$. An asterisk denotes that the concatenation result is worse than the individual result.

Name	Classifier								Name	Classifier							
	SVM				RF					SVM				RF			
	Acc	Spe	Sen	AUC	Acc	Spe	Sen	AUC		Acc	Spe	Sen	AUC	Acc	Spe	Sen	AUC
[AO]	86.7	*84.0	88.0	90.6	87.3	82.0	90.0	89.7	[AOV]	90.7	82.0	95.0	95.0	91.3	92.0	91.0	95.1
[AV]	88.0	*76.0	94.0	94.5	90.7	90.0	91.0	95.0	[AOVv]	91.3	88.0	93.0	93.9	93.3	92.0	94.0	94.1
[AVv]	93.3	90.0	95.0	94.1	92.7	90.0	94.0	93.9	[AOR]	92.0	90.0	93.0	94.1	*86.7	*82.0	*89.0	91.5
[AR]	92.0	90.0	93.0	94.1	88.7	88.0	89.0	91.3	[AOI]	88.7	86.0	90.0	92.4	93.3	84.0	98.0	91.7
[AI]	88.0	86.0	89.0	92.1	90.7	82.0	95.0	91.7	[AVVv]	93.3	88.0	96.0	97.2	93.3	90.0	95.0	96.8
[OV]	90.0	82.0	94.0	94.8	92.0	90.0	93.0	94.8	[AVR]	93.3	84.0	98.0	96.8	92.0	88.0	94.0	95.8
[OVv]	90.7	90.0	91.0	94.1	92.0	92.0	92.0	94.6	[AVI]	92.0	86.0	95.0	94.3	92.7	80.0	99.0	93.5
[OR]	92.0	90.0	93.0	94.3	89.3	88.0	90.0	93.2	[AVvR]	93.3	90.0	95.0	94.2	92.7	90.0	94.0	93.8
[OI]	87.3	84.0	89.0	91.3	90.7	86.0	93.0	91.5	[AVvI]	90.0	88.0	91.0	93.1	91.3	86.0	94.0	93.5
[VVv]	90.7	88.0	92.0	95.9	93.3	90.0	95.0	97.5	[ARI]	88.7	86.0	90.0	92.9	90.7	84.0	94.0	92.2
[VR]	92.0	86.0	95.0	95.4	90.0	86.0	92.0	93.3	[OVVv]	92.0	88.0	94.0	97.3	93.3	90.0	95.0	97.7
[VI]	87.3	80.0	91.0	93.4	*88.7	78.0	94.0	92.9	[OVR]	94.7	88.0	98.0	97.4	93.3	94.0	93.0	96.4
[VvR]	88.7	90.0	88.0	92.1	*84.7	*88.0	*83.0	91.5	[OVI]	90.7	82.0	95.0	94.4	91.3	82.0	96.0	94.7
[VvI]	*84.0	*80.0	*86.0	89.4	86.7	*82.0	*89.0	90.4	[OVvR]	90.7	90.0	91.0	94.2	91.3	90.0	92.0	95.3
[RI]	85.3	82.0	87.0	89.4	85.3	78.0	89.0	87.0	[OVvI]	88.7	*84.0	91.0	92.5	91.3	86.0	94.0	93.0
									[ORI]	88.0	84.0	90.0	92.2	88.7	*80.0	93.0	90.4
									[VVvR]	92.0	88.0	94.0	96.4	93.3	90.0	95.0	97.1
									[VVvI]	90.0	*84.0	93.0	94.8	92.7	*86.0	96.0	95.8
									[VRI]	88.0	*82.0	91.0	94.2	90.0	*78.0	96.0	91.3
									[VvRI]	86.7	*84.0	88.0	90.2	87.3	*82.0	*90.0	91.0

**Table 6:** Feature concatenation (4 or more CNNs) results: $[{{\rm{N}}}_{1},\cdots ,{{\rm{N}}}_{{\rm{k}}}],\,{\rm{k}}=4,5,6$ denotes feature concatenation using the features from CNNs, ${{\rm{N}}}_{1}$ to ${{\rm{N}}}_{{\rm{k}}}$. An asterisk denotes that the concatenation result is worse than the individual result.

Name	Classifier							
	SVM				RF			
	Acc	Spe	Sen	AUC	Acc	Spe	Sen	AUC
[AOVVv]	93.3	88.0	96.0	96.8	94.0	92.0	95.0	97.1
[AOVR]	93.3	84.0	98.0	96.9	92.7	92.0	93.0	95.6
[AOVI]	92.7	84.0	97.0	94.7	92.7	84.0	97.0	94.5
[AOVvR]	92.0	90.0	93.0	94.1	92.7	90.0	94.0	94.1
[AOVvI]	91.3	86.0	94.0	92.9	91.3	86.0	94.0	92.8
[AORI]	89.3	86.0	91.0	92.9	91.3	*84.0	95.0	91.4
[AVVvR]	94.0	90.0	96.0	97.3	92.7	*88.0	95.0	97.4
[AVVvI]	91.3	88.0	93.0	95.8	92.0	*88.0	94.0	94.6
[AVRI]	93.3	86.0	97.0	95.0	92.0	*84.0	96.0	93.8
[AVvRI]	90.0	88.0	91.0	93.3	92.0	*86.0	95.0	93.4
[OVVvR]	93.3	90.0	95.0	97.4	94.0	94.0	94.0	98.5
[OVVvI]	90.0	*84.0	93.0	95.2	91.3	*86.0	94.0	96.2
[OVRI]	92.0	84.0	96.0	95.0	92.0	*78.0	99.0	94.4
[OVvRI]	90.0	88.0	91.0	93.1	92.0	*88.0	94.0	93.4
[VVvRI]	90.0	*84.0	93.0	95.4	90.0	*84.0	93.0	94.4
[AOVVvR]	94.0	90.0	96.0	96.9	93.3	90.0	95.0	97.0
[AOVVvI]	93.3	90.0	95.0	95.7	92.7	*88.0	95.0	95.5
[AOVRI]	93.3	86.0	97.0	95.2	93.3	86.0	97.0	94.2
[AOVvRI]	92.0	88.0	94.0	93.2	92.7	*88.0	95.0	94.6
[AVVvRI]	91.3	88.0	93.0	95.9	92.0	*88.0	94.0	94.1
[OVVvRI]	92.0	88.0	94.0	95.8	90.7	*86.0	93.0	95.5
[AOVVvRI]	93.3	90.0	95.0	95.7	90.7	*86.0	93.0	95.6

### Classification ensemble

In this subsection, the same features which were named as [A],[O],[V],[Vv],[R], and [I] in the previous section were used again. We first executed a classification ensemble of SVM and RF results with single CNN-based features and these results are written in italic, *[A]*,*[O]*,*[V]*,*[Vv]*,*[R]*, *and [I]*. To compare these with individual results (the results found using features from a single CNN) in Table 3, we defined $\hat{J}$ as follows

$$\hat{J}=\frac{{\rm{Acc}}/{\rm{Spe}}/{\rm{Sen}}\,{\rm{with}}\,{\rm{classification}}\,{\rm{ensemble}}}{{\rm{maximum}}\,{\rm{of}}\,{\rm{Acc}}/{\rm{Spe}}/{\rm{Sen}}\,{\rm{with}}\,{\rm{single}}\,{\rm{CNN}}\,{\rm{feature}}\,{\rm{and}}\,{\rm{classifier}}\,{\rm{SVM}}/{\rm{RF}}\,{\rm{in}}\,{\rm{Table}}\,2}.$$

The value $\hat{J}$ is an indicator of the performance of the classification ensemble. An asterisk (*) was used to mark performance values of classification ensembles that had a $\hat{J}$ smaller than 1. As shown in Table 7, several hierarchical steps of the classification ensemble affected overall accuracies while the classification ensemble of SVM and RF for a single CNN did not improve accuracies significantly.

**Table 7:** Classification ensemble results: $[{M}_{1},\cdots ,{M}_{k}]$ denotes classification ensemble, where [${M}_{i}$] indicates the ensemble result of SVM and RF using ${M}_{i}$ CNN-based features. An asterisk denotes that the classification ensemble result is worse than the individual result.

Name	Acc	Spe	Sen	AUC	Name	Acc	Spe	Sen	AUC	Name	Acc	Spe	Sen	AUC
*[A]*	*86.0	82.0	88.0	88.5	*[A][O][V]*	90.7	90.0	91.0	94.2	*[A][O][V][Vv]*	93.3	90.0	95.0	96.6
*[O]*	*86.0	84.0	*87.0	88.8	*[A][O][Vv]*	91.3	90.0	92.0	94.3	*[A][O][V][R]*	93.3	88.0	96.0	96.6
*[V]*	*87.3	*82.0	*90.0	90.6	*[A][O][R]*	89.3	90.0	*89.0	93.5	*[A][O][V][I]*	93.3	88.0	96.0	94.7
*[Vv]*	*83.3	*88.0	*81.0	90.7	*[A][O][I]*	89.3	86.0	91.0	91.4	*[A][O][Vv][R]*	92.0	*88.0	94.0	95.0
*[R]*	*84.7	90.0	*82.0	90.9	*[A][V][Vv]*	92.0	*86.0	95.0	97.6	*[A][O][Vv][I]*	93.3	*88.0	96.0	94.0
*[I]*	*84.0	*70.0	91.0	87.3	*[A][V][R]*	94.7	90.0	97.0	97.6	*[A][O][R][I]*	92.0	86.0	95.0	93.5
*[A][O]*	*84.7	*80.0	*87.0	89.0	*[A][V][I]*	92.7	*84.0	97.0	95.5	*[A][V][Vv][R]*	93.3	*88.0	96.0	97.7
*[A][V]*	90.7	*80.0	96.0	95.2	*[A][Vv][R]*	90.7	90.0	91.0	95.0	*[A][V][Vv][I]*	92.0	*84.0	96.0	96.6
*[A][Vv]*	92.0	*88.0	94.0	94.9	*[A][Vv][I]*	90.7	*86.0	93.0	94.1	*[A][V][R][I]*	94.0	88.0	97.0	96.5
*[A][R]*	93.3	88.0	96.0	94.1	*[A][R][I]*	92.7	86.0	96.0	93.4	*[A][Vv][R][I]*	90.0	*88.0	91.0	94.3
*[A][I]*	90.7	82.0	95.0	91.5	*[O][V][Vv]*	93.3	*88.0	96.0	97.5	*[O][V][Vv][R]*	92.0	90.0	93.0	97.7
*[O][V]*	91.3	86.0	94.0	95.2	*[O][V][R]*	92.7	88.0	95.0	97.5	*[O][V][Vv][I]*	92.7	*86.0	96.0	96.8
*[O][Vv]*	90.7	*88.0	92.0	95.1	*[O][V][I]*	94.0	86.0	98.0	95.6	*[O][V][R][I]*	92.0	86.0	95.0	96.6
*[O][R]*	90.7	88.0	92.0	94.6	*[O][Vv][R]*	90.0	90.0	90.0	95.0	*[O][Vv][R][I]*	89.3	88.0	90.0	94.4
*[O][I]*	92.7	84.0	97.0	92.1	*[O][Vv][I]*	89.3	*86.0	91.0	94.4	*[V][Vv][R][I]*	88.7	*86.0	90.0	96.1
*[V][Vv]*	90.0	*88.0	91.0	96.8	*[O][R][I]*	91.3	86.0	94.0	93.7	*[A][O][V][Vv][R]*	94.0	90.0	96.0	97.2
*[V][R]*	92.0	86.0	95.0	97.1	*[V][Vv][R]*	89.3	88.0	90.0	97.2	*[A][O][V][Vv][I]*	94.0	90.0	96.0	96.2
*[V][I]*	89.3	*76.0	96.0	94.2	*[V][Vv][I]*	88.7	*82.0	92.0	96.0	*[A][O][V][R][I]*	94.0	88.0	97.0	96.1
*[Vv][R]*	88.7	92.0	87.0	91.5	*[V][R][I]*	92.0	*82.0	97.0	96.0	*[A][O][Vv][R][I]*	91.3	*88.0	93.0	94.6
*[Vv][I]*	86.7	*86.0	87.0	91.4	*[Vv][R][I]*	87.3	*86.0	88.0	91.6	*[A][V][Vv][R][I]*	93.3	*88.0	96.0	97.0
*[R][I]*	86.0	*84.0	*87.0	90.9						*[O][V][Vv][R][I]*	90.7	*88.0	92.0	96.9
										*[A][O][V][Vv][R][I]*	92.0	*86.0	95.0	96.6

### Combination of feature concatenation and classifier ensemble

A combination of the two previously proposed approaches was also performed. For the feature concatenation, we used the results of Table 6 and then we applied the classification ensemble of SVM and RF results. As seen in Tables 7 and 8, feature concatenation plays a key role while the classifier ensemble merely affects the results.

**Table 8:** Results for when both feature concatenation and classifier ensemble were performed.

Name	Acc	Spe	Sen	AUC	Name	Acc	Spe	Sen	AUC
[AOVVv]	93.3	88.0	96.0	97.0	[OVVvI]	91.3	86.0	94.0	95.9
[AOVR]	93.3	82.0	99.0	96.6	[OVRI]	92.0	78.0	99.0	95.0
[AOVI]	93.3	84.0	98.0	94.8	[OVvRI]	92.0	88.0	94.0	93.3
[AOVvR]	92.0	90.0	93.0	94.3	[VVvRI]	90.0	84.0	93.0	95.2
[AOVvI]	92.0	86.0	95.0	93.1	[AOVVvR]	93.3	88.0	96.0	97.1
[AORI]	91.3	84.0	95.0	92.3	[AOVVvI]	93.3	88.0	96.0	95.9
[AVVvR]	94.0	90.0	96.0	97.5	[AOVRI]	92.7	84.0	97.0	94.9
[AVVvI]	92.0	88.0	94.0	95.5	[AOVvRI]	92.7	88.0	95.0	93.8
[AVRI]	93.3	84.0	98.0	94.4	[AVVvRI]	92.0	88.0	94.0	95.3
[AVvRI]	92.0	86.0	95.0	93.5	[OVVvRI]	90.7	86.0	93.0	95.7
[OVVvR]	93.3	90.0	95.0	98.0	[AOVVvRI]	90.7	86.0	93.0	96.0

### Diagnostic performances of radiologists and CNNs

The diagnostic performances of the 6 radiologists and 3 CNN-combinations for the diagnosis of thyroid malignancy are shown in Table 1. We chose three types of CNN-based feature concatenations and classifier ensembles from Tables 6 and 7 which were shaded. CNN 1 stands for the results obtained from trained SVM using features from [A], [V], [Vv] and [R]. CNN 2 represents RF classifier results trained with [O], [V], [Vv] and [R]-based features. CNN 3 corresponds to the results from the ensemble outcome of SVM and RF which were both trained with concatenated features from [O], [V] and [I]. Experienced radiologists showed higher accuracies than less experienced radiologists (Table 1). Compared to the diagnostic performances of the two experienced radiologists, differences in accuracies were not statistically significant (*P* = 0.309). Faculty 1 showed significantly higher sensitivity than faculty 2 (*P* < 0.001). In contrast, faculty 2 showed significantly higher specificity than faculty 1 (*P* = 0.006). Accuracies of faculty 1, faculty 2, CNN 1, CNN 2, and CNN 3 were 78%, 82.7%, 94%, 94%, and 94%. Accuracies of the 3 CNNs were significantly higher than those of the 2 faculties (Table 1). Specificities of the 3 CNNs were significantly higher than that of faculty 1 (90% of CNN 1, 94% of CNN 2, 86% of CNN 3 vs 52% of faculty 1, *P* < 0.001) (Table 9). Sensitivities of the 3 CNNs were significantly higher than that of faculty 2 (96% of CNN 1, 94% of CNN 2, 98% of CNN 3 vs 76% of faculty 2, *P* < 0.001) (Table 9).

**Table 9:** Comparisons of diagnostic performances between experienced radiologists and CNNs for thyroid malignancy.

	Accuracy	Specificity	Sensitivity
Faculty1 vs Faculty2	0.309	<.001	0.006
Faculty1 vs CNN1	<.001	<.001	0.163
Faculty1 vs CNN2	<.001	<.001	0.424
Faculty1 vs CNN3	<.001	<.001	0.046
Faculty2 vs CNN1	0.004	0.257	<.001
Faculty2 vs CNN2	0.004	0.649	<.001
Faculty2 vs CNN3	0.003	0.102	<.001

### Interobserver variability and agreement of US assessments for predicting thyroid malignancy among 6 radiologists and between 2 radiologists with similar experience levels

Interobserver agreement to diagnose thyroid malignancy among the 6 radiologists was 0.465, which meant a moderate degree of agreement Table 10). Interobserver agreements for the differentiation of thyroid nodules was 0.387 (fair agreement) for the two faculties, 0.663 (substantial agreement) for the two fellows, and 0.418 (moderate agreement) for the two residents.

**Table 10:** Interobserver variability for the prediction of thyroid malignancy among 6 radiologists and between 2 radiologists with similar levels of experience.

Radiologist	Kappa (95% CI)
All	0.465 (0.388, 0.535)
Faculties	0.387 (0.226, 0.511)
Fellows	0.663 (0.540, 0.784)
Residents	0.418 (0.286, 0.557)

## Discussion

We have proposed a CADx system which can provide reliable supplementary and objective information to help radiologists in the decision-making process. More precisely, we focused on constructing an efficient and accurate CADx system for thyroid US image classification using deep learning and this was achieved by concatenating features extracted from various pre-trained CNNs and training classifiers based on those features. Six pre-trained CNNs, AlexNet, OverFeat, VGG, VGG-verydeep, ResNet, and Inception, were utilized in feature extraction and two classifiers, SVM and RF, were used. In the overall process, 594 training and 150 test images were used. Table 2 shows that the results of pre-trained CNNs with fine-tuning were not much better than those of the radiologists (Table 1). A past study^38^ also found similar results. The pre-trained CNN, VGG-F, was utilized to classify the US images of thyroid nodules. The study only focused on using a single CNN to determine the label of each test image.

Our approach suggested using pre-trained CNNs only for feature extraction and training them with SVM or RF classifiers. More importantly, we proposed combining features from various CNNs (feature concatenation) and combining the results from different classifiers (classifier ensemble). The different structures of various CNNs allow the creation of different features, which motivates our approach. Several factors have to be considered before CNN-based feature extraction is used in CADx: CNN selection, performance of fine-tuning, extracted layer selection, and classifier selection. We conducted all possible combinations considering these factors and the results are reported in this paper. The pre-trained CNNs, AlexNet, OverFeat, VGG, and VGG-verydeep have two feature extractable layers in which self-feature-concatenation was possible. But, it turned out that self-feature-concatenation was not very effective. In AlexNet, OverFeat, and VGG, feature extraction with fine-tuning led to results with higher accuracy. On the contrary, for VGG-verydeep, ResNet, and Inception, the results of feature extraction without fine-tuned CNNs were generally better than those obtained with fine-tuned CNNs. Since these latter three CNNs have deeper layers, we supposed that the extracted features from the original pre-trained CNNs were sufficiently objective and fine-tuning may have degraded meaningful features instead.

When a single CNN was used to extract features, the results (Table 3) were almost the same with fine-tuned pre-trained CNNs in Table 2. Moreover, a classifier ensemble using features from a single CNN (from the first to the sixth row in the first column of Table 7) had results that were not that different from those obtained without a classifier ensemble, as can be seen in Table 3. Based on the results in Tables 5–7, we conclude that feature concatenation with more CNNs produces better results while a classifier ensemble does not.

When the diagnostic performances of the 6 radiologists and 3 CNN-combinations were analyzed, accuracies of the 3 CNN-combinations (all 94%) were significantly higher than those (78% and 82.7%) of the 2 experienced radiologists. Specificities were significantly higher with the 3 CNN-combinations (86%~94%) than that (52%) of faculty 1. The 3 CNN-combinations (94%~98%) also had significantly higher sensitivities than that (76%) of faculty 2. Furthermore, the interobserver agreement for the final assessment among the 6 radiologists was fair (κ = 0.387) for the 2 faculties, substantial (κ = 0.663) for the 2 fellows, moderate (κ = 0.418) for the 2 residents, and moderate (κ = 0.465) for all 6 radiologists (2 faculties, 2 fellows, and 2 residents). Therefore, a CADx system using CNN-combinations may help radiologists make decisions by overcoming interobserver variability when assessing thyroid nodules on US.

In our opinion, feature concatenation with many CNNs shows promising performance and we expect this approach to be a potential supplementary tool for radiologists. In the future, we plan to examine the proposed method with more data and with medical images from other devices such as MR and CT. Another aiming challenge is developing an efficient localization scheme using concatenating methodology. Our research has excluded the localization task since all US images in this study had a square region-of-interest (ROI) that was depicted by experienced radiologists (with all US images being either cytologically proven or sugically confirmed). There has been research on a CNN-based framework conducting both detection and classification. For example, a multi-task cascade CNN framework was proposed^39^ to detect and recognize nodules and the framework was able to fuse different scales of features in a single module. This spatial pyramid module seems promising as a detection and classification scheme can be established with features from multiple CNNs.

## Methods

### Patients

Institutional review board (IRB) approval was obtained for this retrospective study and the requirement for informed consent for review of patient images and medical records was waived. The patients in the current cohort^38^ had been included in a previous study that used a computerized algorithm to predict thyroid malignancy with a deep CNN to differentiate malignant and benign thyroid nodules on US. Unlike previous studies, we separated the feature extraction and classification processes to enhance the efficiency and accuracy of the previously studied deep CNN algorithms. Multiple deep CNNs were only used for feature extraction and conventional machine learning algorithms were applied for classification.

From May 2012 to February 2015, 1576 consecutive patients who underwent US and subsequent thyroidectomy were recruited. Of those, 592 small nodules from 522 patients were excluded because they were microcalcifications. Finally, we included 589 small nodules equal to or larger than 1 cm and less than 2 cm on US from 519 patients (426 women and 93 men, 47.5 years ± 12.7). The mean size of the 589 nodules was 12.9 mm ± 2.5 (range, 10–19 mm). All of the nodules were confirmed by histopathological examination after surgical excision. Of the 396 malignant nodules, 376 (94.9%) were conventional papillary thyroid carcinoma (PTC), 14 (3.5%) were the follicular variant of PTC, 4 (1%) were the diffuse sclerosing variant of PTC, 1 (0.3%) was the Warthin-like tumor variant of PTC, and 1 (0.3%) was a minimally invasive follicular carcinoma. For the 193 benign nodules, 154 (80%) were adenomatous hyperplasia, 25 (13%) were lymphocytic thyroiditis, 8 (4%) were follicular adenoma, 2 (1%) were Hurthle cell adenoma, 2 (1%) were hyaline trabecular tumors, 1 (0.5%) was a hyperplastic nodule, and 1 (0.5%) was a calcific nodule without tumor cells. We designated 439 (142 benign and 297 malignant) US images as the training dataset and 150 (50 benign and 100 malignant) US images as the test dataset. To balance the training set, data augmentation was applied for the benign training data by left-right flipping and up-down flipping so that 155 additional benign images were added to the training dataset. As a result, a total of 594 US images were used as the training data and 150 US images were used as the test data. All US images were labeled as benign or malignant and cropped by a ROI.

### Image acquisition

One of 12 physicians dedicated to thyroid imaging performed US with a 5-to 12-MHz linear transducer (iU22; Philips Healthcare, Bothell, WA) or a 6–13-MHz linear transducer (EUB-7500; Hitachi Medical, Tokyo, Japan). A representative US image was obtained for each tumor considering US findings by K.J.Y who had 16 years of experience in analyzing thyroid US images. The images were stored as JPEG images in the picture archiving and communication system. Square regions of interest (ROIs) were drawn using the Paint program of Windows 7.

### Image analyses

**Figure 1:**
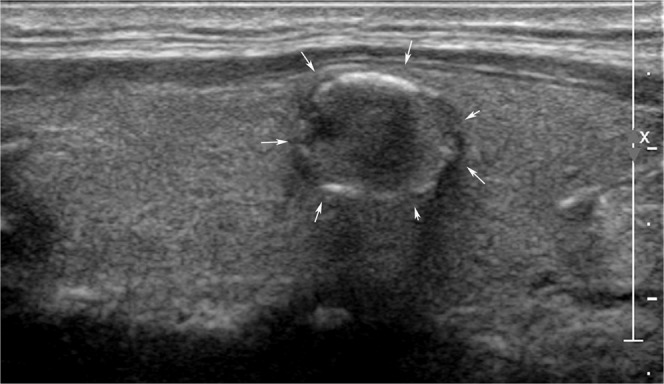
An ultrasonography (US) image of a 50-year-old woman with an incidentally detected thyroid nodule discovered on screening examination that shows a 1.2-cm sized hypoechoic solid nodule with eggshell calcifications (arrows). All 6 radiologists interpreted the nodule as a benign. In contrast, 3 CNN-combinations interpreted it as cancer. The nodule was diagnosed as papillary thyroid cancer by surgery.

**Figure 2:**
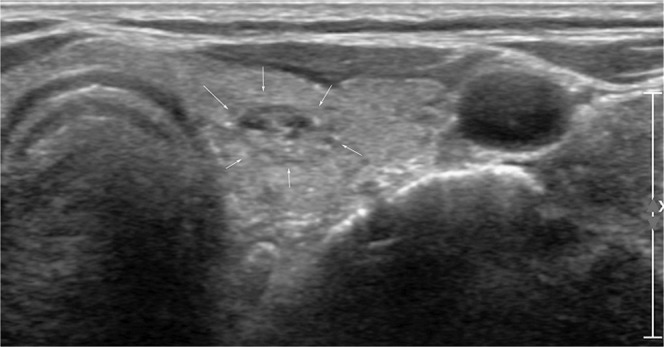
An ultrasonography (US) image of a left thyroid nodule in a 77-year-old woman who was confirmed with cancer in the right thyroid gland. A 1-cm sized isoechoic nodule with internal echogenic spots was seen (arrows). Four radiologists (1 faculty, 1 fellow, and two residents) interpreted the nodule as cancer. In contrast, 3 CNN-combinations interpreted it as benign. The nodule was diagnosed as adenomatous hyperplasia.

A total of 150 US images (50 benign and 100 malignant) were reviewed by two faculties (K.H.J. and M.H.J.) with 8 and 16 years of experience in thyroid imaging, two second-year fellows (B.J.H. and H.S.), and two second-year residents (S.J.W. and Y.J.), retrospectively. Each physician categorized the nodules as ‘probably benign‘ or ‘suspicious malignant‘ based on the criteria from Kim et al.^40^. which classified a nodule as ‘suspicious malignant‘ when any of the suspicious US features (markedly hypoechogenicity, microlobulated or irregular margins, microcalcifications, and taller-than-wide shape) were present. In Figs. 1 and 2, two clinical cases were introduced.

### Feature extraction using pre-trained CNN

**Figure 3:**
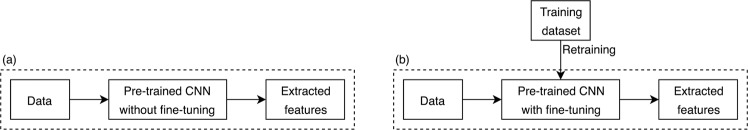
Two feature extraction strategies using pre-trained CNN: Feature extraction from pre-trained CNN without fine-tuning (**a**) or with fine-tuning (**b**).

In CNN, high-level features were generated as images passed through multiple layers. Here, two different approaches were used when the features were extracted. One was to collect the generalized (or objective) features from pre-trained CNNs directly (Fig. 3(a)). The other was to train pre-trained CNNs with modifications of the last layer to fit the given data (Fig. 3(b)). In this process, pre-trained parameters were considered as initial information and these parameters were fine-tuned by the training dataset so that they would carry information about the given training data. The overall procedure was transfer learning and fitted features were extracted from fine-tuned CNNs. The pre-trained CNNs, AlexNet^32^, OverFeat-accurate^33^, VGG-F^34^, VGG-19^35^, ResNet-50^36^, and Inception-v3^37^, were used to extract features.

### Feature concatenation

**Figure 4:**
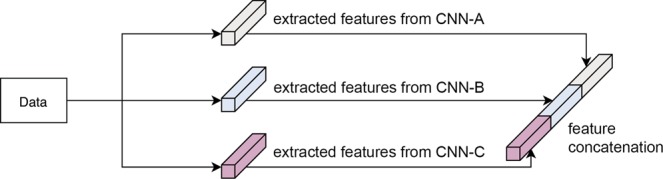
Example of feature concatenation: Feature concatenation of features extracted from three different CNNs.

Features extracted from deeper layers are compressive, so discriminative information may have been missed. Also, different CNNs might have differentiated information. While some CNNs (AlexNet, VGG, VGG-verydeep) had several possible feature extractable layers, the others had only one feature layer to extract features. To catch the sensible information, we examined features extracted from different layers in a particular CNN and those from different CNNs in various combinations. For instance, Fig. 4 describes the feature concatenation of features extracted from three different CNNs.

### Classification ensemble

Once a feature set was ready, a classifier was trained to establish the results. We employed two classifiers, SVM and RF, to produce results with different criteria. The two classifiers may agree but sometimes they give conflicting results. To observe an objective result, we applied the classification ensemble by averaging the results from the classifiers as follows.

**Figure 5:**
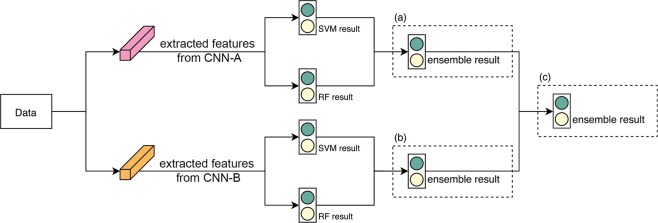
Example of classification ensemble: Two CNNs were used as feature extractors and then classification ensembles were applied for SVM and RF of CNN-A(**a**) and CNN-B(**b**) to observe results. For further objective results, the classification ensemble was again applied for ensemble results(**c**).

For a given input image **x**, let ${{\rm{f}}}_{{\rm{r}}}({\bf{x}}),\,r=1,\cdots ,R$be trained with classifiers and let ${{\bf{p}}}_{{\rm{r}}}=\{{p}_{(r,0)},\,{p}_{(r,1)}\}$ be the output of ${{\rm{f}}}_{{\rm{r}}}({\bf{x}})$, where ${p}_{(r,0)}$ and ${p}_{(r,1)}$ are the probabilities that the feature in question corresponds respectively to benign and malignant. Then, the outputs from each classifier were averaged to generate new probability results ${\hat{p}}_{0}\,$and ${\hat{p}}_{1}$ as follows

$${\hat{p}}_{0}=\frac{1}{R}\mathop{\sum }\limits_{r=1}^{R}{p}_{(r,0)}\,{\rm{and}}\,{\hat{p}}_{1}=\frac{1}{R}\mathop{\sum }\limits_{r=1}^{R}{p}_{(r,1)}$$

and this is the ‘classification ensemble’. In Fig. 5(a,b) delineate the abovementioned process.

This classification ensemble can be extended to cases using multiple feature sets as well. Let $[{{\rm{N}}}_{{\rm{j}}}],\,{\rm{j}}=1,\cdots ,{\rm{M}}$ be the feature set extracted from CNN_j_ (or the feature set obtained from j-th feature concatenation), then ${{\rm{f}}}_{{\rm{r}}}^{j}({\bf{x}})$ is the trained classifier using features in $[{{\rm{N}}}_{{\rm{j}}}]$ and ${{\bf{p}}}_{{\rm{r}}}^{j}=\{{p}_{(r,0)}^{j},\,{p}_{(r,1)}^{j}\}$is the corresponding probability result. Then, a more objective result can be obtained by averaging the ensemble results through the procedure below

$$\begin{array}{c}{\hat{p}}_{0}^{1}=\frac{1}{R}\mathop{\sum }\limits_{r=1}^{R}{p}_{(r,0)}^{1}\,{\rm{and}}\,{\hat{p}}_{1}^{1}=\frac{1}{R}\mathop{\sum }\limits_{r=1}^{R}{p}_{(r,1)}^{1}\,\\ \vdots \\ {\hat{p}}_{0}^{M}=\frac{1}{R}\mathop{\sum }\limits_{r=1}^{R}{p}_{(r,0)}^{M}\,{\rm{and}}\,{\hat{p}}_{1}^{M}=\frac{1}{R}\mathop{\sum }\limits_{r=1}^{R}{p}_{(r,1)}^{M}\end{array}\}\Rightarrow {\hat{p}}_{0}=\frac{1}{R}\mathop{\sum }\limits_{j=1}^{M}{\hat{p}}_{0}^{j}\,{\rm{and}}\,{\hat{p}}_{1}=\frac{1}{R}\mathop{\sum }\limits_{j=1}^{M}{\hat{p}}_{1}^{j}$$

The above approach does not require any additional training processes because the ensemble method only uses results already obtained. Figure 5 illustrates classification ensembles with SVM and RF using two feature sets.

### Data and statistical analysis

To evaluate the performances of radiologists and CNNs for predicting thyroid malignancy, sensitivity, specificity, and accuracy with 95% confidence intervals were estimated and compared with the logistic regression using the generalized estimating equation. We calculated the interobserver variability. Fleiss’s kappa statistics were used for interobserver variability among the 6 radiologists and Cohen’s kappa statistics were used for interobserver variability between the two radiologists with similar levels of experience. To obtain 95% confidence intervals of kappa statistics, the bootstrap method was used with resampling done 1000 times. We interpreted kappa statistics as follows: 0.01–0.20 (slight agreement), 0.21–0.40 (fair agreement), 0.41–0.60 (moderate agreement), 0.61–0.80 (substantial agreement) and 0.81–0.99 (almost perfect agreement^41^).

*P* values less than 0.05 were considered statistically significant. Data analysis was performed using R version 3.5.1 (R Foundation for Statistical Computing, Vienna, Austria).

## Supplementary information

Supplementary Information
