# Supplementary material for: Differentiation of thyroid nodules on US using features learned and extracted from various convolutional neural networks
Source: Sci Rep. 2019 Dec 27;9:19854. doi: 10.1038/s41598-019-56395-x (PMC6934479; doi:10.1038/s41598-019-56395-x)
Supplement: Supplementary file 1 — Supplementary Information [file 41598_2019_56395_MOESM1_ESM.docx]

**Supplementary Information**

**Differentiation of thyroid nodules on US using features learned and extracted from various convolutional neural networks**

Eunjung Lee, Ph.D.^1*^, Heonkyu Ha^1^, Hye Jung Kim, M.D., Ph.D.^2^, Hee Jung Moon, M.D., Ph.D.^3^, Jung Hee Byon, M.D.,Ph.D.^3,^ Sun Huh, M.D.^3^, Jinwoo Son, M.D.^3^, Jiyoung Yoon, M.D.^3^, Kyunghwa Han, PhD^3^, Jin Young Kwak, M.D., Ph.D.^3*^

^1^Department of Computational Science and Engineering, Yonsei University, ^2^Department of Radiology, School of Medicine, Kyungpook National University, Kyungpook National University Chilgok Hospital, ^3^Department of Radiology, Severance Hospital, Research Institute of Radiological Science, Yonsei University College of Medicine

Corresponding author 1: Eunjung Lee, Ph.D.

Department of Computational Science and Engineering, Yonsei University

50 Yonsei-ro, Seodaemun-gu, 120-752 Seoul, Korea

TEL: 82-2-2123-6128, FAX: 82-2-2123-8194, E-mail: [eunjunglee@yonsei.ac.kr](mailto:eunjunglee@yonsei.ac.kr)

Corresponding author 2: Jin Young Kwak M.D., Ph.D.

Department of Radiology, Research Institute of Radiological Science, Yonsei University College of Medicine

50 Yonsei-ro, Seodaemun-gu, 120-752 Seoul, Korea

TEL: 82-2- 2228-7413, FAX: 82-2-393-3035, E-mail: [docjin@yuhs.ac](mailto:docjin@yuhs.ac)

***Pre-trained CNNs used in feature extraction***

In ILSVRC, almost 1.2 million images with 1000 classes are used to train nets. Here we briefly introduce the CNNs that were used in this paper.

AlexNet^32^ was the winner of the ILSVRC 2012. AlexNet outperformed previous results which used handcrafted features and provided the motivation to focus on deep learning. AlexNet was inspired by LeNet and was expanded to a large scale dataset with GPU computing. It uses the Rectified Linear Unit (ReLU) as a nonlinear activation function to overcome the gradient-vanishing problem and uses data augmentation and the dropout technique to prevent overfitting. AlexNet greatly impacted CNN models, especially ZFNet which won the ILSVRC 2013 by modifying AlexNet's hyperparameters. In this paper, we extracted features from two fully-connected layers.

OverFeat^33^ was the winner of localization at the ILSVRC 2013. Like ZFNet, OverFeat is similar to AlexNet. The difference is that OverFeat uses non-overlapping max pooling. Although OverFeat did not show the best performance for classification at the ILSVRC 2013, Razavian *et al.* showed that using OverFeat as a feature extractor can be powerful. There are two models of OverFeat, fast and accurate. Here, the accurate model was used and we extracted features from two fully-connected layers.

VGG^34^ networks were designed by modifying OverFeat, ZFNet, and AlexNet and named as VGG-S, VGG-M, and VGG-F, respectively. The networks investigated the effect of image augmentation, color information, fine-tuning, and low dimensional features for CNNs. In this paper, VGG-F was used and we extracted features from two fully-connected layers.

VGG-19^35^ models have 11-19 layers by using smaller convolutional filters. To improve the performance of CNNs, the VGG team investigated the effect of deeper CNNs. This experiment was successful in showing the advantages of deeper CNNs and its findings affected the development of many CNNs thereafter. In this paper, VGG-19 was used and we extracted features from two fully-connected layers.

ResNet^36^ was designed by Microsoft and the winner of classification task at the ILSVRC 2015. ResNet introduced residual learning that concatenated the output of two convolutional layers and bypassed input. Residual learning made networks much deeper than VGG-verydeep without increasing training errors. To decrease complexity, ResNet did not use hidden fully-connected layers and dropout techniques. As a result, the 34-layer ResNet computes faster than the 19-layer VGG-verydeep even though ResNet is deeper. We used ResNet-50 and extracted features from one global average pooling layer.

Inception^37,41,42^ was designed by Google. Previous CNNs used single type convolutional filters but Inception introduced the inception module that uses various convolution filters and concatenated them to construct much more complex networks. To reduce computational costs and maintain effects, 1x1 convolutional blocks were used. This technique supported the computational costs of inception modules so that the number of parameters of the recent Inception models are much smaller than VGG-19. In this paper, Inception-v3^37^ was used to extract features of one global average pooling layer.

***Classification***

We considered SVM^43^ and RF^44^ as classifiers. The training dataset T is defined as $T=\left\{ \left( \mathbf{x}_{\mathbf{i}}, y_{i} \right):\mathbf{x}_{\mathbf{i}}\in\mathbf{R}^{\alpha}, y_{i}\in\left\{ 0,1 \right\}, i=1,\cdots,N \right\},$where $\mathbf{x}_{\mathbf{i}}$ is input, $y_{i}$ is output, $N$ is the number of training data and $\alpha$ is the feature dimension of training data. Trained classifiers produced probability outputs for test data and we adopted the higher probability for determination.

- Support vector machine (SVM)

Here, we briefly explain the classification problem of SVM with two classes. SVM aims to find the hyperplane which separates two different classes. Generally, there is no unique hyperplane if two classes are perfectly separated. To set the hyperplane uniquely, one gives the constraint that the margin, the distance of data with other classes divided by a hyperplane, is the maximum. Unfortunately, most problems may not be separated perfectly. There are several ways to accommodate these cases, including using the soft margin and kernel trick in which the soft margin seeks a hyperplane to maximize the margin and separate the data as much as possible and kernel trick transforms data into another dimension that has a clear dividing margin between data classes using the kernel function. The detailed usage of the kernel function and the mechanisms of SVM can be found in a previous publication on SVM ^43^. In this paper, we use SVM with soft margin and RBF kernel function.

- Random forests (RF)

The decision tree is a widely known classifier, but is easily overfitted (high variance) when decision trees are complicated. The bootstrap aggregating (bagging) method is one ensemble method used to reduce variance and is often applied for the decision tree. The idea of bagging is to generate *L* training datasets $\left\{ T_{l} \right\}_{l=1}^{L}$ by random sampling with a replacement for *T*. A decision tree can be constructed for each $T_{l}$, and then the ensemble method is operated by majority voting from the classification result of each decision tree. For each decision tree, some training data may not be selected and the unchosen data are called ‘out-of-bag’ data. Thus, out-of-bag data can act as a validation dataset so that an optimal *L* can be chosen. The bagging method can reduce variance, but co-variances are also considered because each decision tree is not independent. RF^44^ is an extension of the bagging method used to decrease the co-variances of the bagging method. The overall process of RF is similar to the bagging method, but each decision tree uses training data with a lower feature dimension $m<M$ by random selection without replacement from *M* features. Generally *m* is chosen as$\sqrt{M}$.

***Feature selection***

Feature selection^45,46^ chooses a subset of original features that has less effects and ambiguous properties. We used a simple feature selection method for binary classification^47^

$$D_{k}=\left| \frac{1}{N_{benign}}\sum_{y_{i}=benign} x_{ik}-\frac{1}{N_{malignant}}\sum_{y_{i}=malignant} x_{ik} \right| , k=1,\cdots,N,$$

where the training data have ${N=N}_{benign}+N_{malignana}$ data with two classes $\left\{ \left( \mathbf{x}_{\mathbf{i}}, y_{i} \right):y_{i}\in\left\{ benign,malignant \right\}, i=1,\cdots,N \right\}$. We also tested all the experiments with a feature selection process by selecting the top 50% or 75% of features. However, the feature selection process had little effect on the results; that is, the results were very similar or sometimes they got worse. Therefore, we decided not to report the results obtained with feature selection in this paper.

***Implementation***

As addressed, we used 6 pre-trained CNNs (AlexNet, OverFeat-accurate, VGG-F, VGG-19, ResNet-50, and Inception-v3). Each network was pre-trained by ImageNet and fine-tuned by the given training data. To fine-tune each CNN, the stochastic gradient descent with momentum (SGDM) was used as an optimizer with mini-batch 33, momentum 0.9, and cross-entropy loss function. Optimal epochs, learning rate, and learning decay for each CNN were selected by 6-fold cross-validation. Supplementary Table A summarizes the fine-tuning information for each CNN. In classifier training, SVM with soft margin and RBF kernel function was implemented by MATLAB R2018a with the function *fitcsvm* and hyperparameters $\gamma$ and *C* were optimized by using grid search with 6-fold cross-validation. RF was implemented by MATLAB R2018a with the function *TreeBagger* and hyperparameter *L* was optimized by using the out-of-bag error.

Supplementary Table B describes the hardware specifications used in implementation. The computational time for each fine-tuning process of CNN was up to 6 seconds per epoch when GPU computing was applied. This training speed per epoch in CNN mainly depends on the number of parameters, so deeper CNNs require much more time. OverFeat was different because it was trained with CPU computing and it took 2 minutes per epoch. When the feature concatenation of 6 CNNs were applied, the computational times of SVM and RF were up to 7 seconds with fixed hyperparameters. Note that depicted computational times may vary depending on the performance of the computer.

**Supplementary Table A.** CNN fine-tuning information

| Net | Language  (version) | Package  (version) | Pre-trained weights | Epoch  (ep) | Learning  Rate | Learning decay |
| --- | --- | --- | --- | --- | --- | --- |
| AlexNet | MATLAB  (R2018a) | Neural Network (NN) Toolbox | NN Toolbox | 7 | 1e-03 | 0.1 for every 5 ep |
| OverFeat | Lua  (5.3.2) | Torch7 | NYU CILVR lab | 20 | 1e-03 | decreasing log-scale to 1e-04 |
| VGG | MATLAB  (R2018a) | MatConvNet  (1.0-beta25) | MatConvNet | 12 | 1e-03 | 0.1 for every 5 ep |
| VGG-verydeep | Python  (3.6.5) | Keras(2.1.6)  Tensorflow(1.7.0) | Keras | 54 | 1e-05 | - |
| ResNet | Python  (3.6.5) | Keras(2.1.6)  Tensorflow(1.7.0) | Keras | 37 | 1e-04 | - |
| Inception | Python  (3.6.5) | Keras(2.1.6)  Tensorflow(1.7.0) | Keras | 9 | 1e-03 | 0.1 for every 5 ep |

**Supplementary Table B.** Hardware specifications

| Info | OS | CPU/GPU |
| --- | --- | --- |
| AlexNet | Windows | Nvidia GTX 1050 Ti (GPU Computing) |
| VGG |  |  |
| OverFeat | Mac | Intel i7-7700K (CPU Computing) |
| SVM |  |  |
| RF |  |  |
| VGG-verydeep | Linux | Nvidia GTX 1080 Ti (GPU Computing) |
| ResNet |  |  |
| Inception |  |  |
